# Low doses of 3-aminobenzamide, a poly(ADP-ribose) polymerase inhibitor, stimulate angiogenesis by regulating expression of urokinase type plasminogen activator and matrix metalloprotease 2

**DOI:** 10.1186/2045-824X-3-12

**Published:** 2011-05-19

**Authors:** Riccardo Caldini, Elena Fanti, Lucia Magnelli, Emanuela Barletta, Elisabetta Tanganelli, Michele Zampieri, Marta Chevanne

**Affiliations:** 1 Department of Experimental Pathology and Oncology, University of Florence, viale G.B. Morgagni 50, 50134 Florence, Italy; 2 Department of Cellular Biotechnologies and Haematology, II Faculty of Medicine and Surgery, "Sapienza" University of Rome, viale Regina Elena 324, 00161 Rome, Italy

## Abstract

**Background:**

Poly(ADP-Ribose) polymerase (PARP) activity has been demonstrated fundamental in many cellular processes, including DNA repair, cell proliferation and differentiation. In particular, PARP activity has been recently found to affect proliferation, migration, and tube formation of human umbilical vein endothelial cells. In recent times, PARP inhibitors have entered in clinical trials to potentiate cancer treatments by preventing DNA repair, but little is known about the effects performed by different drug concentrations on neoangiogenesis, an essential step in tumor growth.

**Methods:**

Human umbilical vein endothelial cells were treated with 3 aminobenzamide (3ABA), a PARP inhibitor, and tested for several different cellular parameters.

**Results:**

Here we present *in vitro *evidence that a low concentration of 3ABA (50 μM), stimulates angiogenesis by decreasing fibrinolytic activity, carried out by urokinase-type plasminogen activator (uPA), and by enhancing matrix metalloprotease-2 (MMP-2) gelatinolytic activity, in fibroblast growth factor-2-stimulated endothelial cells. These unbalanced pathways modify *in vitro *angiogenic steps, inhibiting chemoinvasion and stimulating tubulogenic activity.

**Conclusions:**

Our results suggest that the proangiogenic effect of low concentrations of 3ABA alerts on the efficacy of PARP inhibitors to potentiate anticancer therapy. Moreover, they indicate that endothelial chemoinvasion and tubulogenesis depend on distinct proteolytic pathways.

## Background

Angiogenesis, the process of formation of new blood vessels from preexisting capillaries, is essential for normal development in embryos. In adults, with some exceptions like the female reproductive cycle and the granular tissue in wound healing processes, angiogenesis is an undesired process in certain pathological conditions: in rheumatoid arthritis, in psoriasis, in diabetic retinopathy, in the enlarging atherosclerosis plaque, and in other pathologic phenomena including cancer [1]. In particular, the growth of capillaries into tumors leads to their enlargement and helps the tumor cells to metastasize. Angiogenesis requires endothelial cells to migrate, proliferate, and ultimately assemble into tubes that regulate selective transport of blood cells and solutes from their lumen to the interstitium and vice versa.

Endothelial cell survival and proliferation are a prerequisite for migration, sprouting and tubulogenesis. Endothelial cell proliferation is stimulated by a number of soluble factors and cytokines including fibroblast growth factor (FGF). The basic form (FGF2) is known to be a strong migration, sprouting, survival, and proliferation factor for endothelial cells, and plays a key role in both normal blood vessel formation and in pathologic angiogenesis. Neovascularization, however, requires endothelial cells to accomplish a number of other tasks such as matrix degradation and migration to appropriate sites.

It is well known that the serine protease urokinase-type plasminogen activator (uPA) and the metalloproteases (MMPs) regulate endothelial cell migration and adhesion during angiogenesis. The uPA system is involved in angiogenesis, cancer progression, invasion as well as tumor prognosis [2]. This system is important for cell-associated proteolytic activity that regulates cell migration and adhesion through binding of uPA to the uPA receptor (uPAR) and subsequent extracellular matrix degradation. MMPs participate not only in the remodeling of basement membrane and extracellular matrix (ECM), but also contribute to angiogenesis by releasing ECM-bound growth factors, and by exposing cryptic, proangiogenic integrin binding sites in the ECM [3].

Recently, we found that, in transformed endothelial GM7373 cells, FGF2-dependent uPA upregulation is affected by poly(ADP-ribose) polymerase (PARP) activity stimulated by MAPK-dependent phosphorylation [4,5]. Poly(ADP-ribosyl)ation is a posttranslational modification of proteins involved in most cellular functions. A central role of PARP family enzymes (PARPs) was demonstrated in many fundamental processes, including cell proliferation and differentiation, by regulating gene expression via posttranslational modification of gene regulating proteins and histones [6]. Beneficial effects of various PARP inhibitors have been demonstrated in several forms of endothelial dysfunction [7,8]. Several PARP inhibitors are currently undergoing clinical evaluation [9,10], and a toxic effect against non proliferating cells was hypothesized [11]. Pharmacological inhibitors of PARP affect proliferation, migration, and tube formation of human umbilical vein endothelial cells [12-14], but little is known about the molecular mechanisms involved in these antiangiogenic effects. Moreover, some compounds that inhibit tumor growth at high concentrations can stimulate tumor growth at lower concentrations, and an hormetic dose-response curve is observed [15]. Several angiogenesis inhibitors have been reported to produce a biphasic dose-efficacy: statins [16], safrole oxide [17] and natural products such as α-bisabolol [18]. To our knowledge, the effect of low doses PARP inhibitors on angiogenesis *in vitro *has not been previously explored.

In this study we were interested to elucidate whether 3ABA, one of the most utilised competitive inhibitor of PARP, used at a low non toxic concentration (50 μM), could affect migratory and tubulogenic capability of endothelial cells, and if it could alter uPA/uPAR and MMPs activities.

## Methods

### Cell cultures

Human umbilical vein endothelial cells (HUVEC) and endothelial cell culture media were purchased from Clonetics (Cambrex, Walkersville, MD, USA). Cells were grown on gelatin-coated plastic, in Endothelial cell Growth Medium-2 (EGM-2,) supplemented with 10% FCS (Euroclone, Milan, Italy), at 37°C in 5% CO_2 _humidified atmosphere, and split at a 1:3 ratio for every passage. Cells from the 3rd to 8th passage were used. All assays were performed on subconfluent cell monolayers. Before the experiments the cells were incubated for 24 h in EGM-2 medium containing 2% FCS (starvation medium). 3ABA (Sigma Chemical Co., St. Louis, MO, USA) was added to the medium, at a final concentration of 50 μM, 30 min before the supplementation of FGF2 (10 ng/ml; Boehringer, Mannheim, Germany). Cells were detached with a solution of 0.05% trypsin and 0.02% EDTA in phosphate buffered saline (PBS).

### Chemoinvasion assay

The Boyden chamber technique was performed to evaluate spontaneous (chemokinesis) and stimulated invasion (chemoinvasion) through growth factor-reduced Matrigel matrix (BD Bioscience, Bedford, MA, USA). Polyvinylpyrrolidone-free polycarbonate filters (Neuro Probe, Inc, Gaithersburg, MD, USA) with a pore size of 8 μm were coated with Matrigel (50 μg), allowed to polymerize at 37°C for 60 min, and than placed over the bottom chamber. Cells (2 × 10^5^) from subconfluent cultures were suspended in starvation medium (150 μl) and loaded into the upper chamber. The effect of 3ABA was analyzed by incubating the cells in the presence of 3ABA (50 μM) as previously indicated. For the chemoinvasion test, a solution containing FGF2 (10 ng/ml) as chemo-attractant was placed in the lower chamber. At the end of the experimental time (8 h) the filters were fixed in methanol. Non-migrated cells were removed from the upper side of the filter with a cotton swab, while migrated cells, adherent on the lower filter surface, were stained with Diff-Quik (Mertz-Dade AG, Dade International, Milan, Italy). The chemoinvasive response was determined by counting the migrated cells attached to the lower surface of the filter in 10 randomly selected microscopic fields.

### *In vitro *tubulogenic assay

The formation of vascular-like structures by endothelial cells was assessed on growth factor-reduced Matrigel. In brief, 500 μg of Matrigel were added to each well of 96-well tissue culture plates and allowed to polymerize at 37°C for 60 min. To examine the effect of PARP inhibitor 3ABA on *in vitro *tubulogenesis, HUVEC, cultured in starvation medium for 24 h, were supplemented for 30 min with 3ABA (50 μM) and Ilomastat (25 μM; Calbiochem, La Jolla, CA, USA) or anti-MMP-2 antibody (10 μg/ml; Cell Signaling Technology, Beverly, MA, USA), detached and seeded (1.5 × 10^4 ^cells/200 μl) on Matrigel, in the presence of the above mentioned substances and of FGF2 (10 ng/ml). Cultures were monitored for up to 24 h and photographed after 4 h with an inverted microscope (Leitz DM-IRB) equipped with a CCD camera. Three photographic fields from three plates were scanned for each point. Results were quantified by measuring the percent field occupancy of tubules using ImageJ 1.45b software (National Institutes of Health, Bethesda, MD, USA).

### Cell-associated plasminogen activator (PA) activity assay

To assay cell-associated PA activity cells were harvested, washed twice with PBS and extracted on ice with 0.5% Triton X-100 in 60 mM Tris-HCl pH 8.5 (T/T buffer). Proteins (5 μg) were incubated in a microtiter plate with 2 μg of purified human plasminogen (Boehringer, Mannheim, Germany) and 25 μg of the plasmin chromogenic substrate Chromozym PL (Tosyl-glycyl-prolyl-lysine-4-nitranilide-acetate) (Boehringer, Mannheim, Germany) in a final volume of 350 μl of T/T buffer. After incubation at 37°C, the plate was read every 15 min at 405 nm wavelength with an automatic microplate reader (Biorad, Hercules, CA, USA). Human uPA (Serono, Roma, Italy) was used as standard.

### Total cytoplasmic RNA extraction and semi-quantitative RT-PCR

Total cytoplasmic RNA (RNA_TC_) was extracted from endothelial subconfluent culture according to the RNeasy protocol (Qiagen GmbH, Hilden, Germany), and single stranded cDNA was synthesized from 1 μg of RNA_TC_. In order to quantify the amount of uPA and uPAR gene mRNA, a semi-quantitative polymerase chain reaction (PCR) procedure was set up using the glyceraldehyde-3-phosphate dehydrogenase (GAPDH) as a control house-keeping gene. Sequences of the specific primers were: uPA primers (5'-3') forward AAAATGCTGTGTGCTGCTGACC and reverse CCCTGCCCTGAAGTCGTTAGTG; uPAR primers (5'-3') forward GCCCTGGGACAGGACCTCTG and reverse GCCGAGGCCCCATGAATCAATG; and human GAPDH primers (5'-3') forward CCACCCATGGCAAATTCCATGGCA and reverse TCTAGACGGCAGGTCAGGTCCACC. The amplification was performed for 35 cycles with an annealing temperature of 48°C for uPA and uPAR, of 55°C for GAPDH. Intensity of bands corresponding to the expected size of the amplified cDNA fragments of uPA, uPAR and GAPDH, 704 bp, 612 bp and 598 bp respectively, were quantified by densitometry using the Image J software. Values were then expressed as the mean ± SEM of the ratios between the normalized values of uPA or uPAR, and GAPDH.

### Gelatin zymography

MMP-2 and MMP-9 activity levels in the medium were determined by gelatin zymography. Cell-free aliquots of serum-free culture medium (conditioned medium) were loaded on 8% SDS-PAGE gels containing 1 mg/ml gelatin. The loading volume of each sample was adjusted in proportion to the protein content in the culture well from which the sample was taken, and ranged from 8 to 25 μl. After electrophoresis, the gel was rinsed with 2.5% Triton X-100 to remove SDS and restore enzyme activity, incubated 16-20 h at 37°C in substrate buffer (20 mM Tris-HCl, pH 7.5, 200 mM NaCl, 5 mM CaCl_2_, 0.02% dodecylpolyethyleneglycolether), stained with 0.1% Comassie Brilliant Blue R-250 dye (Sigma Co.) in 40% methanol/7% acetic acid for 1 h, and destained with 40% methanol/7% acetic acid until bands were apparent: the location of gelatinolytic activity was detected as clear bands, and shown in Figure 5a as inverted dark bands for a better visual prominence. HT1080 human fibrosarcoma cell conditioned medium was used as a marker of molecular weight. Arbitrary density of an individual cleavage band was determined by scanning densitometry using ImageJ software.

### Statistical analysis

Data are expressed as mean ± SEM of the indicated number of experiments. Multiple comparisons were performed by the Student-Newman-Keuls test, after demonstration of significant differences among medians by non-parametric variance analysis according to Kruskal-Wallis.

## Results

### Effect of 3ABA on FGF2 induced chemoinvasion and *in vitro *tubulogenesis

HUVEC has been used to evaluate the effects of PARP inhibitor 3ABA on migration and tubular-like networks formation, which represent two important steps in the angiogenic process.

**Figure 1:**
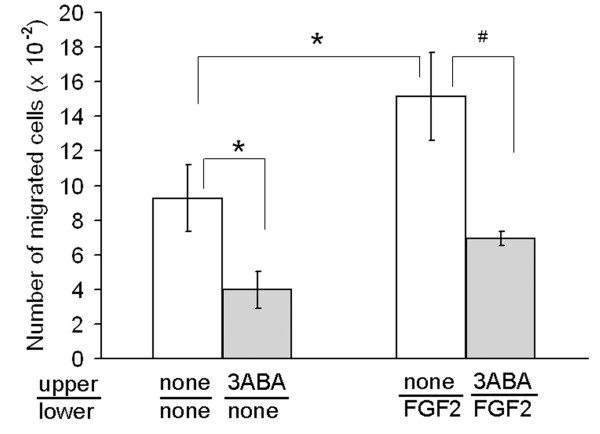
**Effect of 3ABA on *in vitro *chemoinvasion**. Starved HUVEC were plated in the upper Boyden chamber and treated without and with 3ABA (50 μM) for 6 h. FGF2 was used in the lower compartment as chemoattractant. FGF2 induced a significative increase (+63%) of cells ability to invade Matrigel, compared to unstimulated cells. Treatment with 3ABA significantly inhibited both FGF2-stimulated and spontaneous cell invasiveness of about 55%. Values are expressed as the number of cells migrated through pores, and are mean ± SEM of three experiments. Multiple comparisons were performed by the Student-Newman-Keuls test, after Kruskal-Wallis analysis. (* p < 0.05 compared to control; # p < 0.05 compared to FGF2 stimulated cells).

In order to test the ability of a low dose of 3ABA (50 μM) to modulate endothelial cell chemokinesis, and chemoinvasion in response to a migration stimulus, we used an *in vitro *migration assay performed in Boyden chambers (Figure 1). When FGF2 was used as a chemoattractant, it induced a significative increase (+63%) of HUVEC ability to invade Matrigel (* p < 0.05; n = 3) compared to unstimulated cells. Treatment with 3ABA significantly inhibited both FGF2-stimulated (# p < 0.05; n = 3) and spontaneous (* p < 0.05; n = 3) cell invasiveness of about 55%.

**Figure 2:**
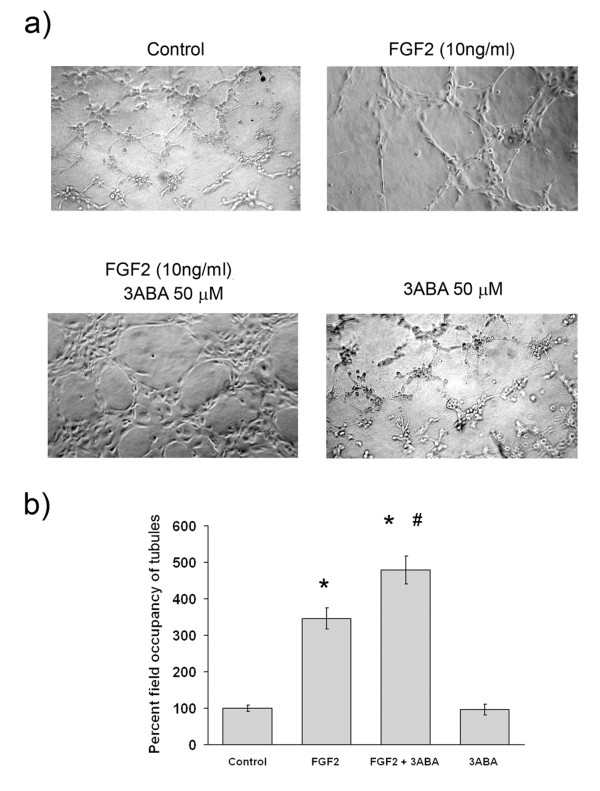
**Effect of 3ABA on *in vitro *tubulogenesis**. **(a) **Representative photographic fields showing the effect of 3ABA on *in vitro *tubulogenesis. HUVEC tubulogenesis assay was performed in the absence (Control), in the presence of FGF2 (10 ng/ml), of FGF2 plus 3ABA (50 μM), and of 3ABA. Pictures were taken after 4 h. HUVEC stimulated with FGF2 started to differentiate in tube-like structures. Exposure to 3ABA of FGF2-stimulated cells enhanced tube formation with a rise of the number of interconnections, and the thickness of tubules. **(b) **Quantification of tubulogenesis after 3ABA treatment. Results were quantified by measuring the percent field occupancy of tubules. Values are mean ± SEM of six experiments. The percent decrease/increase of tubulogenesis is referred to control untreated cells taken as 100%. Multiple comparisons were performed by the Student-Newman-Keuls test, after Kruskal-Wallis analysis. (* p < 0.05 compared to control; # p < 0.05 compared to FGF2 treated cells).

In Figure 2 is reported the effect of 3ABA on *in vitro *tubulogenesis. HUVEC were seeded in 96-well culture plates precoated with the growth factor-reduced Matrigel, and cellular morphology was photographed after 4 h (Figure 2a). The quantification of tubulogenesis by measuring the percent field occupancy of tubules, is reported in Figure 2b. HUVEC cultured in the starvation medium (Control) aggregate in cumuli of rounded cells producing a few distorted cord-like structures. However, when HUVEC were stimulated with FGF2 (10 ng/ml) they started to differentiate in tube-like structures: cells became elongated and formed thin cords of interconnecting cells mimicking the neoangiogenesis process *in vivo *(* p < 0.05; n = 6). Exposure to 3ABA of FGF2-stimulated cells enhanced tube formation with a rise of the number of interconnections, and the thickness of tubules (* p < 0.05, compared to control; # p < 0.05 compared to FGF2 stimulated cells; n = 6), while supplementation of 3ABA alone did not affect cell morphology.

### Effect of 3ABA on cell-associated plasminogen activator (PA) activity

**Figure 3:**
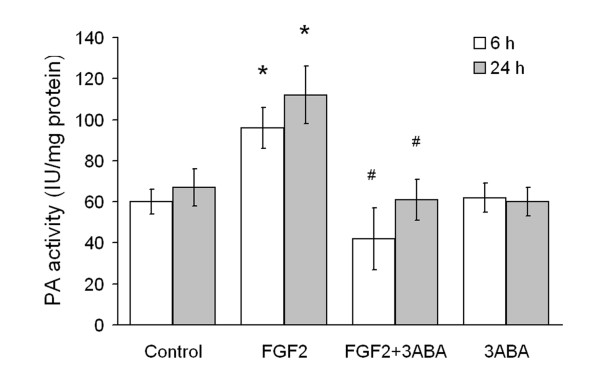
**Effect of 3ABA on cell-associated plasminogen activator (PA) activity**. PA activity was determined spectrophotometrically in untreated HUVEC (Control), and after treatment for 6 and 24 h with FGF2 (10 ng/ml), with FGF2 plus 3ABA (50 μM), with 3ABA. Treatment of cells with FGF2 for 6 and 24 h produced a significative increase in cell-associated PA activity (+60 and 70% respectively) compared to control cells. Values are expressed as International Unit (IU) enzyme/mg protein and are mean ± SEM of four experiments. Multiple comparisons were performed by the Student-Newman-Keuls test, after Kruskal-Wallis analysis. (* p < 0.05 compared to control; # p < 0.05 compared to FGF2 treated cells).

Cell-associated (PA) activity of HUVEC is reported in Figure 3. Cells were cultured in starvation medium (Control) and supplemented with FGF2 (10 ng/ml), in the absence and in the presence of 3ABA. Treatment of HUVEC with FGF2 for 6 h and 24 h produced a significative increase in cell-associated PA activity (+60 and 70% respectively; * p < 0.05 compared to control cells; n = 4). This increase was prevented by 3ABA treatment, maintaining the PA activity at basal levels (# p < 0.05 compared to FGF2 stimulated cells; n = 4). Treatment with 3ABA alone did not modify the basal level of cell-associate PA activity.

### Effect of 3ABA on *uPA *and *uPAR *gene expression

**Figure 4:**
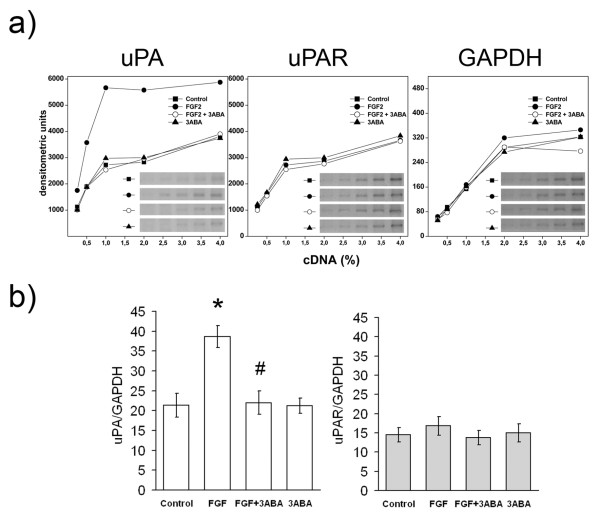
**Effect of 3ABA on *uPA *and *uPAR *gene expression**. **(a) **Representative experiment of *uPA *and *uPAR *gene expression analyzed in HUVEC untreated (Control) and treated with FGF2 (10 ng/ml), with FGF2 plus 3ABA (50 μM), and with 3ABA, for 24 h. cDNAs were serially diluted and amplified by PCR using specific primers for *uPA, uPAR *gene or *GAPDH *as a control housekeeping gene. Cells exposed to FGF2 for 24 h showed an increase in uPA-mRNA compared to control cells, while exposure to 3ABA inhibited FGF2-stimulated *uPA *gene expression. No difference in uPAR-mRNA was found. **(b**) Quantification of *uPA *and, *uPAR *gene expression. Values are expressed as ratio between normalized integrated densities of uPA, uPAR and GAPDH PCR products, and are mean ± SEM of three experiments. Multiple comparisons were performed by the Student-Newman-Keuls test, after Kruskal-Wallis analysis. (* p < 0.05 compared to control; # p < 0.05 compared to FGF2 treated cells).

Since cell associated PA activity induced by FGF2 in endothelial cells is mostly dependent on urokinase type plasminogen activity (uPA) rather than on other proteases [19], we studied the effect of 3ABA on the level of uPA mRNA. The increase of cell-associated PA activity in FGF2-stimulated cells was confirmed by the increased expression of uPA mRNA. Figure 4 shows **(a) **a typical densitometric pattern of uPA, uPAR and GAPDH cDNAs, and **(b) **the quantification of uPA and uPAR cDNAs amplified by PCR, with *GAPDH *as a control house-keeping gene. On the basis of the ratios between normalized values of uPA amplified products and of GAPDH products, we found that cells exposed to FGF2 for 24 h showed a 75% increase of uPA-mRNA (Figure 4b, left side; * p < 0.05 compared to control cells; n = 3). The addition of 3ABA inhibited FGF2-stimulated *uPA *gene expression (# p < 0.05 compared to FGF2 stimulated cells; n = 3), while it did not affect basal levels, confirming results previously obtained in transformed endothelial cells GM7373 [4]. No variation in uPAR mRNA levels was found in the presence of FGF2 nor in the presence of 3ABA, at least after 24 h of treatment (Figure 4b, right side).

### Effect of 3ABA on gelatinolytic activity

**Figure 5:**
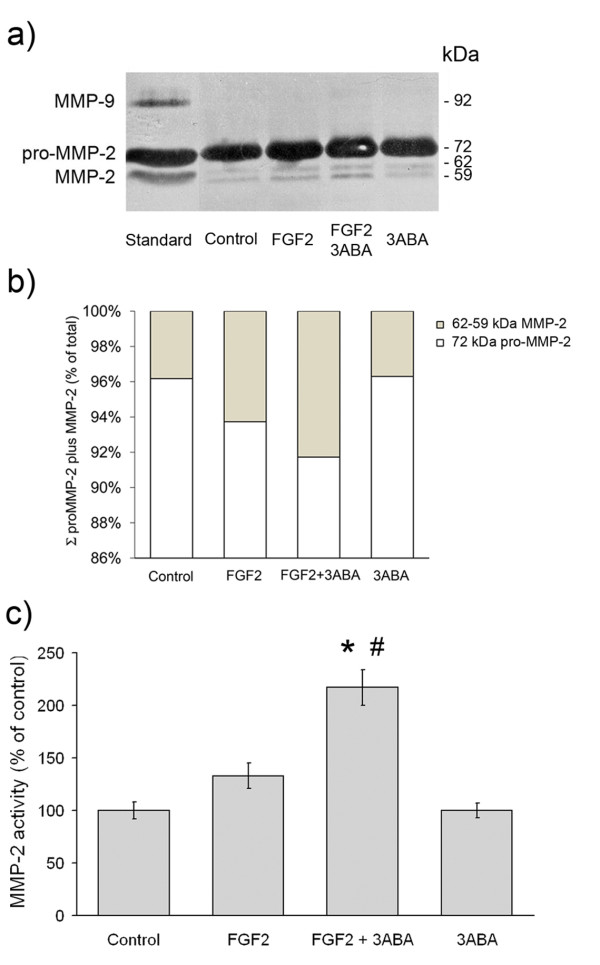
**Effect of 3ABA on MMPs gelatinolytic activity**. **(a) **Representative zymogram, showing MMP-2 and MMP-9 activity in medium from untreated cells (Control), in the presence of FGF2 (10 ng/ml), of FGF2 plus 3ABA (50 μM), and of 3ABA. HT1080 human fibrosarcoma cell conditioned medium was used as a marker of molecular weight. The areas of protease activity appeared as dark bands. **(b) **On the basis of image analysis of zymogram reported in Figure 5a, the percent amount of MMP-2 active form (MMP-2) over the summation (Σ) of pro-MMP-2 plus MMP-2 amounts was calculated. **(c) **Quantification of MMP-2 activity. Densitometric analysis of MMP-2 activity in medium from cells incubated in the absence (Control), in the presence of FGF2, of FGF2 plus 3ABA, and of 3ABA alone. Exposure of FGF2-stimulated HUVEC to 3ABA induced an increase in the active form of MMP-2 compared to both untreated cells (Control), and FGF2 stimulated cells. Values are expressed as percent of control and are mean ± SEM of three experiments. Multiple comparisons were performed by the Student-Newman-Keuls test, after Kruskal-Wallis analysis. (* p < 0.05 compared to control; # p < 0.05 compared to FGF2 treated cells).

Gelatin zymography analysis was utilised to investigate whether MMP activities were modulated by 3ABA treatment in FGF2 stimulated HUVEC. As shown in Figure 5a, HUVEC displayed both the 72 kDa (pro-MMP-2), and the 64 kDa (intermediate MMP-2) and 59 kDa (active MMP-2), forms of the enzyme. They did not express MMP-9 activity. Exposure of FGF2-stimulated cells to 3ABA induced an increase in the active form of MMP-2 compared to both starved cell (Control) and FGF2 cultured cells. No variation in gelatinolytic activity was found in the presence of 3ABA alone, compared to control. On the basis of image analysis of zymogram, the percentage of MMP-2 active form (64 and 59 kDa MMP-2) over the summation of pro-MMP-2 plus MMP-2 amounts (Σ) was calculated. The activities of the MMP-2 active forms, were reported in Figure 5c as percent of the control. Statistical analysis, performed only for the active form, showed a significative increase of MMP-2 activity in FGF2 plus 3ABA treated cells compared to FGF2 stimulated ones (# p < 0.05; n = 3) and to control (* p < 0.05; n = 3).

### Effect of MMP-2 activity inhibition on *in vitro *tubulogenesis

**Figure 6:**
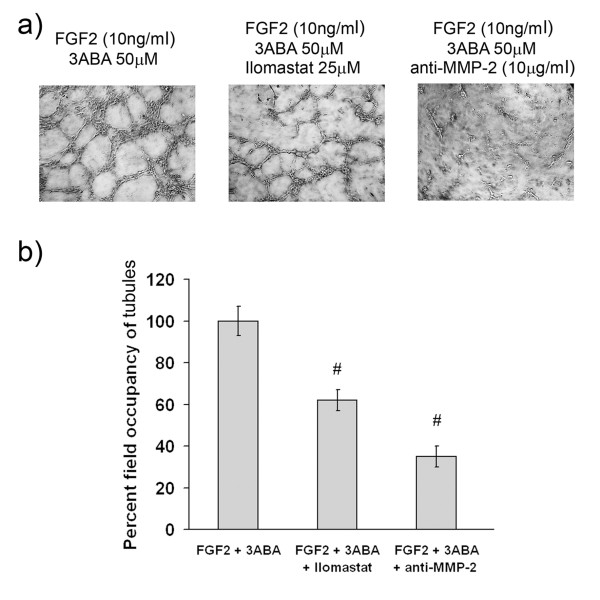
**Effect of MMP-2 inhibition on *in vitro *tubulogenesis**. **(a) **Representative photographic fields showing the effect of MMP-2 inhibition on *in vitro *tubulogenesis of HUVEC treated for 4 h with FGF2 (10 ng/ml) in the presence of 3ABA (50 μM), of 3ABA plus Ilomastat (25 μM), of 3ABA plus anti-MMP-2 antibody (10 μg/ml). **(b) **Quantification of tubulogenesis. Results were quantified by measuring the percent field occupancy of tubules. FGF2-stimulated HUVEC treated with 3ABA decreased the tube formation and the length of the vascular network when supplemented with MMP-2 inhibitor Ilomastat and anti-MMP-2 antibody. Values are mean ± SEM of six experiments. The percent decrease/increase of tubulogenesis is referred to FGF2 plus 3ABA treated cells taken as 100%. Multiple comparisons were performed by the Student-Newman-Keuls test, after Kruskal-Wallis analysis. (# p < 0.05 compared to FGF2 plus 3ABA treated cells).

In order to test whether MMP-2 contributes to the formation of new tubes *in vitro*, we used an unspecific MMPs inhibitor (Ilomastat), and a specific MMP-2 inhibitor (anti-MMP-2 antibody). Representative images of HUVEC cultured on growth factors-reduced Matrigel for 4 h with FGF2 in the presence of 3ABA, of 3ABA plus Ilomastat, of 3ABA plus anti-MMP-2 antibody are reported in Figure 6a. The quantification of the results by measuring the percent field occupancy of tubules is reported in Figure 6b. The length of the vascular network decreased significantly in response to both Ilomastat and anti-MMP-2 antibody (# p < 0.05 compared to FGF2 plus 3ABA stimulated cells; n = 6) These data confirmed the importance of MMP-2 activity in 3ABA stimulated angiogenesis.

## Discussion

The present study shows that treatment of FGF2-stimulated HUVEC with a low dose of 3ABA (50 μM), a well known inhibitor of PARP enzymes, affects angiogenesis leading to inhibition of cell invasion on growth factors-reduced Matrigel and stimulating cells morphogenesis in an *in vitro *tube-forming assay. At the same time, 3ABA stimulates MMP-2 activation, but negatively affects the FGF2-mediated uPA upregulation, inhibiting both protease activity and mRNA amount.

Angiogenesis is a complex process that includes several steps. It begins with the activation of endothelial cells by growth factors, followed by enzymatic degradation of the subendothelial capillary basement membrane by specific proteases, such as uPA and MMPs, allowing endothelial cells to detach from adhesive proteins. Then, cells migrate into the stroma space, proliferate and differentiate into patent structures, by aligning and forming vascular loops. At last, capillary tubes develop with the formation of tight junctions between the cells and the deposition of new basement membrane. Angiogenesis has been generally studied using a variety of approaches: both *in vivo *and *in vitro *models were developed to further explore individual steps and to better understand the molecular mechanisms involved in the angiogenic process. Previous studies from our laboratory have demonstrated that PARP inhibition could affect gene expression of the serine protease uPA by MAPK-dependent pathway, in transformed bovine endothelial cells [4,5]. Given the critical role of uPA and uPAR in angiogenesis and in many human diseases [20,21], it would be of great interest to evaluate the effect of PARP inhibition on some individual steps of angiogenesis which involve both *uPA *and *uPAR *gene expression and MMP-2 activity in a normal human cellular experimental system.

The role of PARPs in angiogenesis has been recently pointed out by several authors [12-14,22,23]. A number of structurally distinct PARP inhibitors (3-aminobenzamide 1-5 mM, PJ34 10-100 μM, 5-aminoisoquinolinone-hydrochloride, and 1,5-isoquinolinediol) showed antiangiogenic effects by inhibiting growth factor expression, or by inhibiting growth factor-induced cellular proliferative responses [23,24], but the direct toxic effect of PARP inhibitors on cellular metabolism must not be disregarded. In fact, high doses (10 μM) of PARP inhibitor PJ34 proved to be cytotoxic on melanoma cells [25]. This cytotoxic effect was evidenced to be independent on DNA damage, but dependent on other housekeeping roles. Moreover, since it reversed when the drug was removed from the medium, it would be of great interest to identify the real concentration of drugs in an *in vivo *system, as well as the pharmacological effects when the concentration of PARP inhibitors fall down inside the microenvironment. In this study we used 3ABA, an extensively studied first generation PARP inhibitor, at a low concentration (50 μM), mainly because at this concentration the drug did not affect viability and proliferative potential of endothelial cells, as well as DNA integrity, but also in order to specifically inhibit poly(ADP-ribosyl)ation without affecting mono ADP ribosylation [26], which has been recently identified as a mechanism for regulating many different aspects of cell physiology [27].

We investigated two important steps of angiogenesis: migration and tubulogenesis *in vitro*, where protease activities are particularly relevant. We found that 3ABA at low doses (50 μM) affects these steps by two mechanisms: i) it dramatically inhibits chemoinvasion, ii) it enhanced differentiation by inducing tube-like structures. Many molecules inhibit separate steps of angiogenesis: plasminogen activator inhibitors block endothelial cell invasion, intracellular signaling inhibitors block endothelial cell migration and proliferation, and matrix metalloprotease inhibitors block tubulogenesis [28,29]. Matrigel invasion assay revealed a marked reduction in both spontaneous and induced chemoinvasion of 3ABA-treated cells compared to control cells. It is likely that such an effect could be related not only to a direct 3ABA effect on any FGF2-independent metabolism, but also to any growth factor constitutively present at a low concentration within the growth factor reduced Matrigel.

It is well known that FGF2-induced endothelial cell migration is uPA mediated. The plasminogen activator system is an important participant in diverse physiological processes including arterial remodelling and angiogenesis. In particular, uPA plays a pivotal role in the regulation of cell migration during tissue remodelling, through proteolysis-dependent and proteolysis-independent mechanisms. The coordinated expression of uPA and uPAR exists at cell-substrate and cell-cell contact sites. The binding of urokinase to uPAR provides both a strictly localized proteolysis of extracellular matrix in the direction of cell movement, and the initiation of a signal transduction cascade, regulating cytoskeleton reorganization, adhesive contact at the leading edge of the cell, and chemotaxis. In this study, 3ABA treatment significantly decreased uPA expression and activity in FGF2-treated cells, and decreased cell movement in response to FGF2 as the chemotactic factor. Collectively, these evidences suggested a relationship between 3ABA treatment and down-regulation of *uPA *gene expression in angiogenic processes, but it cannot be excluded that it might be due to the impaired ability of PARP to control the transcription of specific genes other than *uPA*.

Since the migratory response elicited by FGF2 in bovine aortic endothelial cells was secondary to the induction and autocrine binding of uPA to the uPA receptor, we examined the possibility that uPAR mRNA could be affected too; no difference in uPAR expression was shown in 3ABA cultured cells. Proteases such as uPA and MMPs are key molecules involved in invasion, however the main function of these proteases may not only be the remodelling of the extracellular matrix, but also the processing of a range of molecules involved in angiogenesis. For example, proteases may modify growth factor action by cleaving their receptors. In particular, it is well known that gelatinase MMP-2 is able to cause the shedding of FGF receptor-1. Thus, we investigated the gelatinase activity in 3ABA treated HUVEC. Gelatin zymography of the medium revealed that 3ABA treatment induced endothelial cells to produce small but significant amounts of the cleaved and active MMP-2 form. MMP-2 activation is independent by serine proteases [30] whose activity remained at basal level in FGF2 plus 3ABA-treated HUVEC. High concentration of PARP inhibitors down regulated MMP-2 activity in those experimental systems where angiogenesis resulted inhibited [31], stressing the role of PARP in cellular protease activity regulation.

Moreover, the ability to form a complete tubular network can be strengthened by 3ABA supplementation to a culture medium. Our results are consistent with the study of Schnaper et al. [32], where the formation of tubular networks is increased by the addition of recombinant gelatinase A (MMP-2), and decreased when neutralizing antibodies or the tissue inhibitor of metalloproteases (TIMP) is added. The importance of MMP-2 in tubulogenesis induced by 3ABA was confirmed by MMP-2 neutralization with specific antibody, and with Ilomastat that actually decreased tubulogenesis on Matrigel.

The inhibitory effect on tubulogenesis of high concentration of PARP inhibitors, as reported in the literature [12-14,22,23] seems to disagree with our results: this could be explained by the competition between PARP inhibitors and substrate for binding to MMP-2 [31] that could be ineffective at low doses of inhibitors. A non specific effect on other aspects of cell physiology could be also involved, such as on the mono ADP ribosylation activity [26] that results inhibited by high doses of 3ABA (> 1 mM).

The present study might open a promising perspective in regard to future investigation of PARP inhibitor-dependent modulation of angiogenesis. Further investigations are necessary to clarify the role of PARP on the regulation of gelatinase activation; in particular, it will now be intriguing to understand whether PARP plays a role on the two known pathways involved in the activation of the latent zymogen, namely Membrane Type MMP (MT1-MMP) and Activated Protein C (APC). Moreover, the recently proposed mechanism that links downregulation of PARP and enhanced vascular endothelial growth factor (VEGF) expression [24], caught our attention as it was recently reported that VEGF induced MMP-2 protein activation [33].

## Conclusions

Our study focuses on the effect of PARP inhibitor 3ABA on some important steps of angiogenesis, suggesting the main role of the final concentration reached in the target microenvironment, since very low doses stimulated cells to differentiate, and aggregate in tube-like network, while, as reported in the literature, high doses showed a clear anti angiogenic effect. Furthermore, our results, using FGF2 as exogenous growth factor, suggest that endothelial chemoinvasion and tubulogenesis are dependent on distinct proteolytic pathways: invasion seems to be dependent on uPA activity, while tubulogenesis on MMP-2 gelatinolytic activity.

The proangiogenic effect of low concentrations of the PARP inhibitor 3ABA alerts on the efficacy of PARP inhibitors to potentiate anticancer therapy, and suggests that, in clinical trials, plasma drug concentrations need to be accurately evaluated between administration sessions.

## Competing interests

The authors declare that they have no competing interests.

## Authors' contributions

RC and MC conceived the ideas, performed experiments, analyzed data, produced figures and wrote the paper; ET contributed to work to experiments, EF and LM performed experiments on *in vitro *angiogenesis, EB and MZ performed experiments requiring PCR. All contributed to the writing and the development of the project. All authors have read and approved the final manuscript.
